# Functional analysis of the *rv1371/2/3* operon in mycobacteria

**DOI:** 10.1128/spectrum.03596-25

**Published:** 2026-03-31

**Authors:** Marcos Gustavo Araujo Schwarz, Paloma Rezende Corrêa, Meydson Benjamim Carvalho Corrêa, Antonio Jose da Silva Gonçalves, Livia Carvalho Barbosa, Philip Noel Suffys, Wladimir Malaga, Christophe Guilhot, Leila Mendonça-Lima

**Affiliations:** 1 Laboratório de Biologia Molecular Aplicada à Micobactérias, Instituto Oswaldo Cruz, Fundação Oswaldo Cruz 37903, Rio de Janeiro, Brazil; 2 Laboratório Interdisciplinar de Pesquisas Médicas, Instituto Oswaldo Cruz, Fundação Oswaldo Cruz 37903, Rio de Janeiro, Brazil; 3 Centro de Espectrometria de Massas de Biomoléculas, Universidade Federal do Rio de Janeiro 28125, Rio de Janeiro, Brazil; 4 Centre National de la Recherche Scientifique, Institut de Pharmacologie et de Biologie Structurale 54920, Toulouse, France; University of Minnesota Twin Cities, St. Paul, Minnesota, USA

**Keywords:** *Mycobacterium bovis* BCG, *Mycobacterium tuberculosis*, *rv1371/2/3* operon, lipidomics

## Abstract

*Mycobacterium tuberculosis* is known for its lipid-rich cell wall. Additionally, lipids are also involved in various mycobacterial pathways, such as signaling and energy storage. In this study, using BCG as a model, we aimed to characterize the *rv1371/2/3* operon, which is predicted to be involved in lipid production. Western blotting reveals that single nucleotide polymorphisms in BCG Moreau’s *rv1371* and *rv1373* do not hinder the expression but alter the primary structure of BCG’s Rv1371. Mass spectrometry analysis with the extracted lipids shows that the complemented strains’ (with *Mtb* or BCG alleles) global profiles are quite similar, but slightly differ from those of the knockout (Δ*rv1371/2/*3) in the negative mode. Functionally, Δ*rv1371/2/*3::*Mtb* shows a disadvantage during the early stages of infection in the J774 macrophage model, whereas a significantly higher bacterial load is recovered from cells infected with this strain in later infection stages. Additionally, this strain also shows a higher survival when exposed to SDS compared to the others. These data suggest that this operon may be partially functional in BCG Moreau due to mutations present in the genes, but further experiments are needed to identify the molecule produced from the *Mtb* allele, which leads to the differential phenotype observed in macrophage infection and SDS resistance assays.

**IMPORTANCE:**

This study establishes a functional link between a genetic locus and phenotypic modifications in *Mycobacterium tuberculosis* that may impact the bacillus’s life cycle. We demonstrate that the *rv1371/2/3* operon modulates bacterial lipid composition, which in turn affects macrophage uptake and intracellular persistence. Our findings highlight a functional distinction between the pathogenic *M. tuberculosis* and the attenuated BCG vaccine, providing a model to study how lipid-driven manipulation of host cells alters infection outcomes. Consequently, this operon and its products emerge as potential targets for therapeutic development aimed at disrupting the bacterial mechanisms associated with critical events that culminate in successful infection.

## INTRODUCTION

Tuberculosis remains a significant global health concern, as highlighted by recent data indicating its high mortality rate, ranking it among the leading infectious diseases worldwide (1). Historically, tuberculosis has been strongly associated with inadequate sanitation and poor housing conditions, making it predominantly a poverty-related disease (2). However, with the increasing prevalence of other diseases, such as HIV, the incidence of coinfections has risen. Additionally, the emergence of multidrug-resistant strains of *Mycobacterium tuberculosis* (*Mtb*) has been reported globally. These factors contribute to the reclassification of tuberculosis as a re-emerging infectious disease, particularly in regions where it was previously considered eradicated (3).

The etiological agent of tuberculosis, *M. tuberculosis*, is an obligate aerobe that resides within macrophages during infection, inducing and altering the host’s metabolism to facilitate bacillus development and promote the successful progression of infection (4). This pathogen is recognized for its remarkable competence in the infection process as it can remain viable as an active microorganism even after years of latent infection. Its ability to persist within the host for extended periods, characterized by a reduced metabolic rate and evasion of immune system surveillance, underscores its pathogenic potential. These characteristics are largely encoded in its genome, which contains numerous genes that have been identified as critical at various stages of the infection process (5).

A distinguishing feature of this organism, as well as other mycobacteria, is its complex and large lipid-rich cell wall, which contains several molecules specific to this group, such as mycolic acids (6). In addition to being essential for cell structure, lipids serve as important signaling and storage molecules for mycobacteria (7, 8). Studies have shown that infected macrophages harbor a higher content of lipid droplets in their cytoplasm, which are frequently associated with bacteria-containing vesicles. This observation has led to the hypothesis that these lipid droplets may be utilized by the bacillus for energy storage (9).

Among the important lipids, sulfolipids play a crucial role in the growth and development of mycobacteria (10). As described for *M. tuberculosis*, this microorganism produces several sulfatides, a family of sulfated glycolipids characterized by a common trehalose-2-sulfate core, which is exclusive to the *M. tuberculosis* complex. Among these molecules, sulfolipid I (SL1) is the most extensively studied and is recognized as a significant contributor to strain virulence as its abundance is directly correlated with pathogenicity. Furthermore, SL1 has been shown to modulate host cell responses, including phagosome-lysosome fusion and cytokine production. However, a comprehensive understanding of sulfo-containing molecules’ overall significance in the context of infection and bacteria life cycle remains to be fully elucidated (11).

Not surprisingly, several genes associated with lipid metabolism are mutated in *M. bovis* BCG strains, which are used as live attenuated vaccines against tuberculosis. All these strains are derived from a common *in vitro* attenuation process, followed by several specific evolutionary steps unique to each clade. These evolutionary variations are primarily attributed to differential cultivation and storage protocols employed after the global distribution of the vaccine. Currently, these strains are recognized to harbor multiple genetic synapomorphies that help explain their distinct vaccine characteristics (12). When comparing *M. tuberculosis* with BCG Moreau, the strain used for vaccination in Brazil until 2017, several genomic differences can be observed (13). Our research group has been dedicated to conducting functional genomics studies, correlating such differences with altered phenotypes in the Moreau strain, as demonstrated in the cases of *rv1498A* (14, 15), the fumarate reductase operon (16), and *celA1* (17), among others. Of the other small genomic differences identified, two single nucleotide polymorphisms (SNPs) are identified in genes predicted to encode lipid-metabolism enzymes within a gene organization resembling an operon: the homologs of *rv1371* and *rv1373*. The product of *rv1373* is characterized in *M. tuberculosis* as a glycolipid sulfotransferase; however, there are notable gaps in understanding its native function within the bacillus (18). Additionally, *rv1372*, a gene located in the middle of the operon, encodes a polyketide synthetase (PKS), an enzyme responsible for the elongation and modification of long-chain substrates (19). Given the lack of characterization of this genetic locus and its potential involvement in lipid synthesis and modification (a class of molecules widely recognized as crucial in various contexts of the mycobacterial life cycle), this study will focus on investigating the functional impact of these genetic variations found in BCG Moreau.

To better characterize the function of this predicted operon, we used *M. bovis* BCG Moreau as a model as the described SNPs would result in a non-active or partially active allele compared to that of *M. tuberculosis*. We analyzed the native Rv1371 and Rv1373 homologs using Western blotting to compare changes in protein primary structure, as predicted by gene sequence analysis. Furthermore, we constructed a Δ*rv1371/2/3* knockout strain and complemented both the knockout and wild-type strains with alleles from BCG Moreau and *M. tuberculosis*, allowing us to perform lipid extraction and mass spectrometry (MS) analysis trying to identify differential compounds produced by the complemented strains. To assess the functional role of the *M. tuberculosis* allele, we performed SDS resistance assays, and in the context of infection, we infected J774 macrophages with the generated strains. By compiling all this information, we aim to enhance the understanding of the role of this operon in *M. tuberculosis* and other mycobacteria.

## RESULTS

### Sequence analysis and impact prediction of insertions on *rv1371* and *rv1373* coding sequences

**Fig 1:**
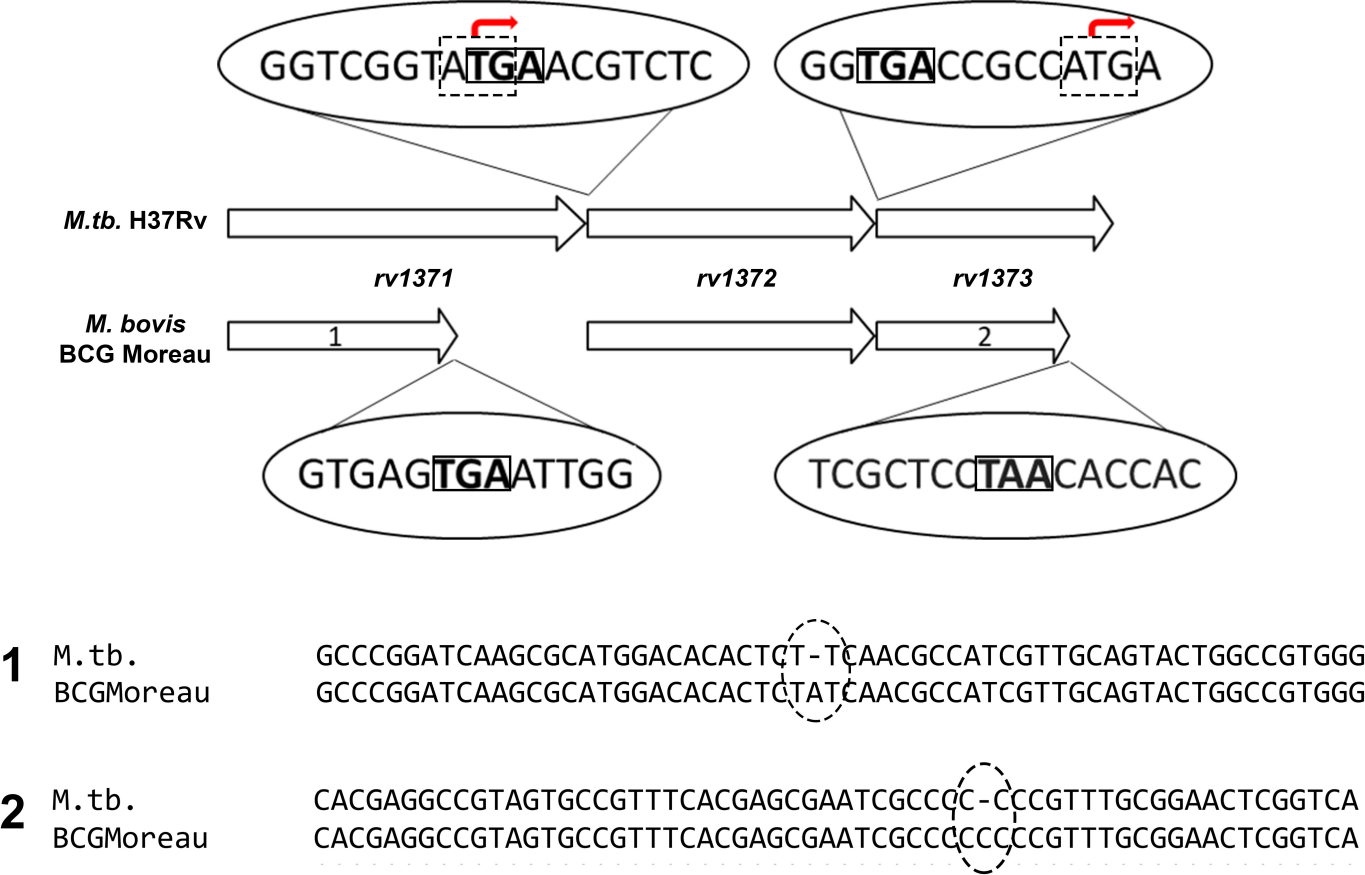
Structures of *M. tuberculosis* and *M. bovis* BCG Moreau *rv1371/2/3* operons. Predicted open reading frame start and stop codons are indicated by dashed-line and solid-line rectangles, respectively. In BCG Moreau, two insertional events are highlighted (dashed-line ellipses): one in *rv1371* (1, an A insertion after T448) and another in *rv1373* (2, a C insertion after C458). The indicated positions correspond to the reference sequences of *rv1371* and *rv1373* from *M. tuberculosis* H37Rv.

As shown in Fig. 1, a comparison between the *M. bovis* BCG Moreau *rv1371* and its homolog in *M. tuberculosis* reveals the presence of an adenine insertion in the former. Similarly, in *rv1373*, a cytosine insertion within a poly-C homopolymer was identified. Both mutations lead to frameshift events, resulting in predicted polypeptides with altered and truncated C-terminal regions (Rv1371: ~33 kDa; Rv1373: ~29 kDa) compared to the corresponding proteins in *M. tuberculosis* (Rv1371: ~55 kDa; Rv1373: 37 kDa). Notably, the *rv1371* mutation exhibits a conserved pattern across all analyzed animal strains within the *M. tuberculosis* complex. The *rv1372* sequences, however, were identical between the two organisms.

Additionally, the stop codon of *rv1371* overlaps with the start codon of *rv1372*, a feature commonly associated with translational coupling. In contrast, in the case of *rv1372* and *rv1373*, there are five nucleotides separating their respective translational start and stop sites.

### Co-transcription of *rv1371/2/3* operon genes and insertions impact on protein structures

**Fig 2:**
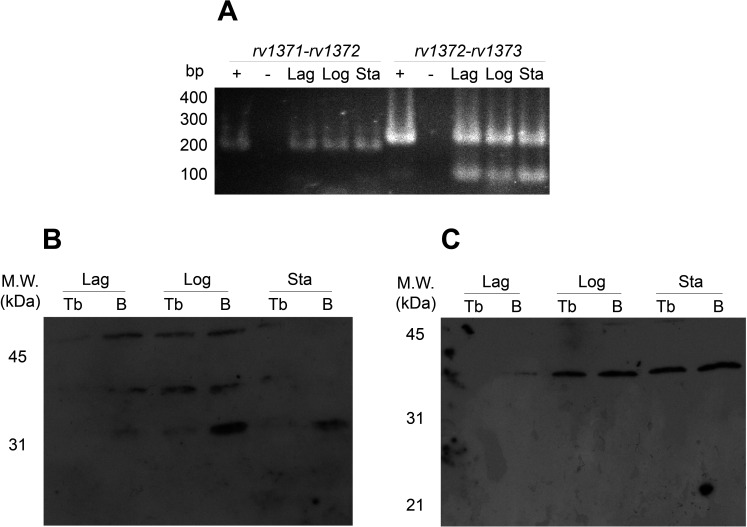
Native characterization of *rv1371/2/3* gene products. (**A**) RT-PCR analysis was performed using intergenic-flanking primer pairs to detect co-transcription of *rv1371-rv1372* and *rv1372-rv1373* during different growth phases: lag, Log, and Sta. Genomic DNA served as the positive control (+), and a no-template reaction was included as the negative control (−). Equal amounts of the RT-PCR product were loaded per lane. (**B**) Rv1371 and (**C**) Rv1373 expressions were assessed in axenic cultures of wild-type *M. tuberculosis* (Tb) and BCG Moreau (B) by Western blotting, using polyclonal sera, across different growth phases.

As shown in Fig. 2A, the studied genes are co-transcribed, as evidenced by the successful amplification of intergenic regions using flanking primers in RT-PCR assays. This result enabled further analysis to investigate the impact of the mutations in *rv1371* and *rv1373* on the expression of their encoded proteins. In Fig. 2B, Rv1371 expression was predominantly observed in samples collected from bacteria in the logarithmic (Log) growth phase, whereas Rv1373 was detected during both the Log and stationary (Sta) phases.

Moreover, for Rv1373, only the *M. tuberculosis* isoform (37 kDa) was observed, both in *M. tuberculosis* and BCG Moreau samples. In contrast, for Rv1371, both isoforms were detected in both microorganisms, with the 33 kDa isoform being more abundant in BCG Moreau.

### Characterization of the functional impacts of the *rv1371/2/3* operon

**Fig 3:**
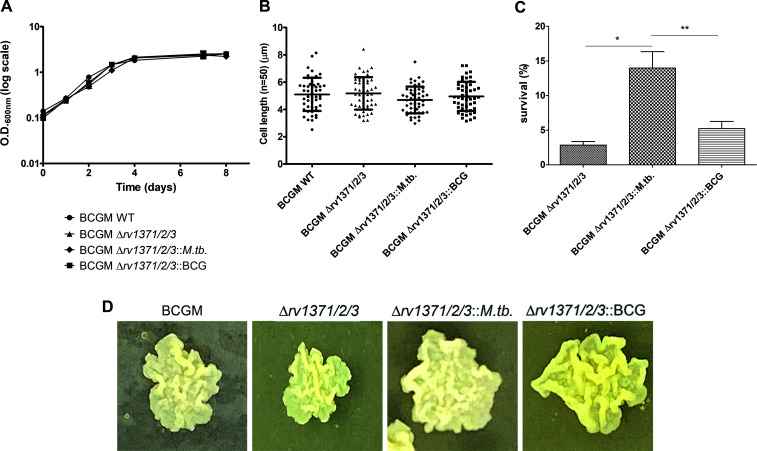
Functional impact of the *rv1371/2/3* operon. (**A**) The growth profile in axenic medium, (**B**) the average size (*N* = 50) of bacterial cells, (**C**) the survival rate after SDS exposure, and (**D**) the colony morphology were analyzed for the different strains. *P < 0.05, ***P < 0.01.

Following the construction of the knockout and complemented strains, initial evaluation of growth in axenic medium was performed. No statistically significant growth differences were observed between the bacterial strains (Fig. 3A), as well as in the average cell length (Fig. 3B) and colony morphology (Fig. 3D). Furthermore, exposure of the different strains to SDS demonstrated that Δ*rv1371/2/3::M.tb*. exhibited a higher survival rate (Fig. 3C).

**Fig 4:**
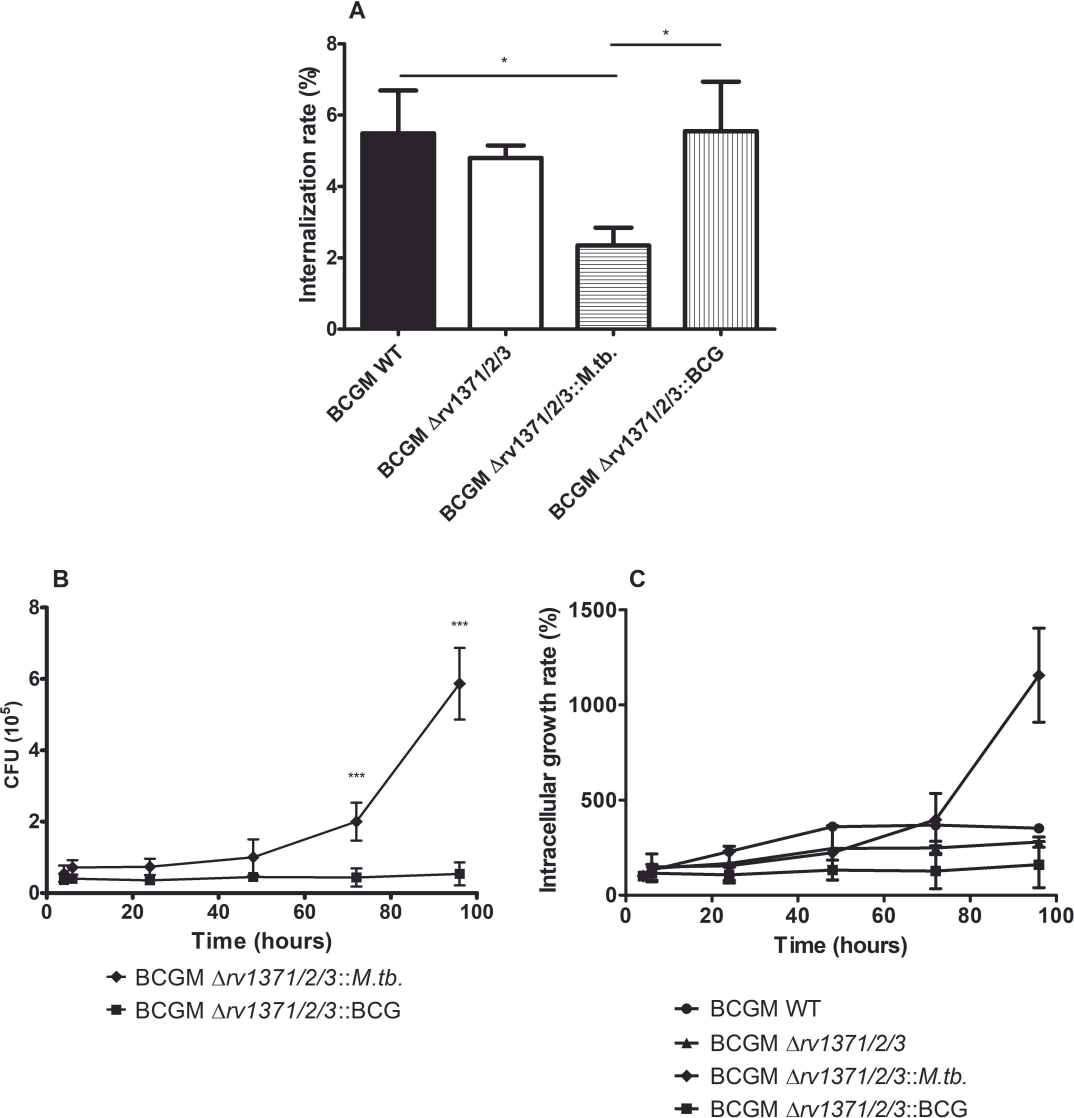
Bacterial growth within macrophages on J774 infection assay using wild-type, *rv1371/2/3* knockout (Δ*rv1371/2/3*), and complemented (Δ*rv1371/2/3::M.tb*. and Δ*rv1371/2/3*::BCG) strains. (**A**) Internalization rate (**B**) and bacterial growth were analyzed. We also evaluated (**C**) the growth rate of all tested strains relative to the 4-h time point. *P < 0.05, **P < 0.001.

After conducting infection assays on the J774 macrophage lineage, we observed, as illustrated in Fig. 4A, that the Δ*rv1371/2/3::M.tb*. strain exhibited a lower internalization rate compared to wild-type, Δ*rv1371/2/3,* and Δ*rv1371/2/3*::BCG strains. However, the growth curve analysis within host cells revealed that the Δ*rv1371/2/3::M.tb*. demonstrated a higher bacterial recovery during the late stages of *in vitro* infection (Fig. 4B). This observation is associated with an increased growth rate (colony-forming unit [CFU] for each time point expressed as a percentage of the 4-h time point value) of this strain during this period of analysis (Fig. 4C).

### Lipidomic analysis

**Fig 5:**
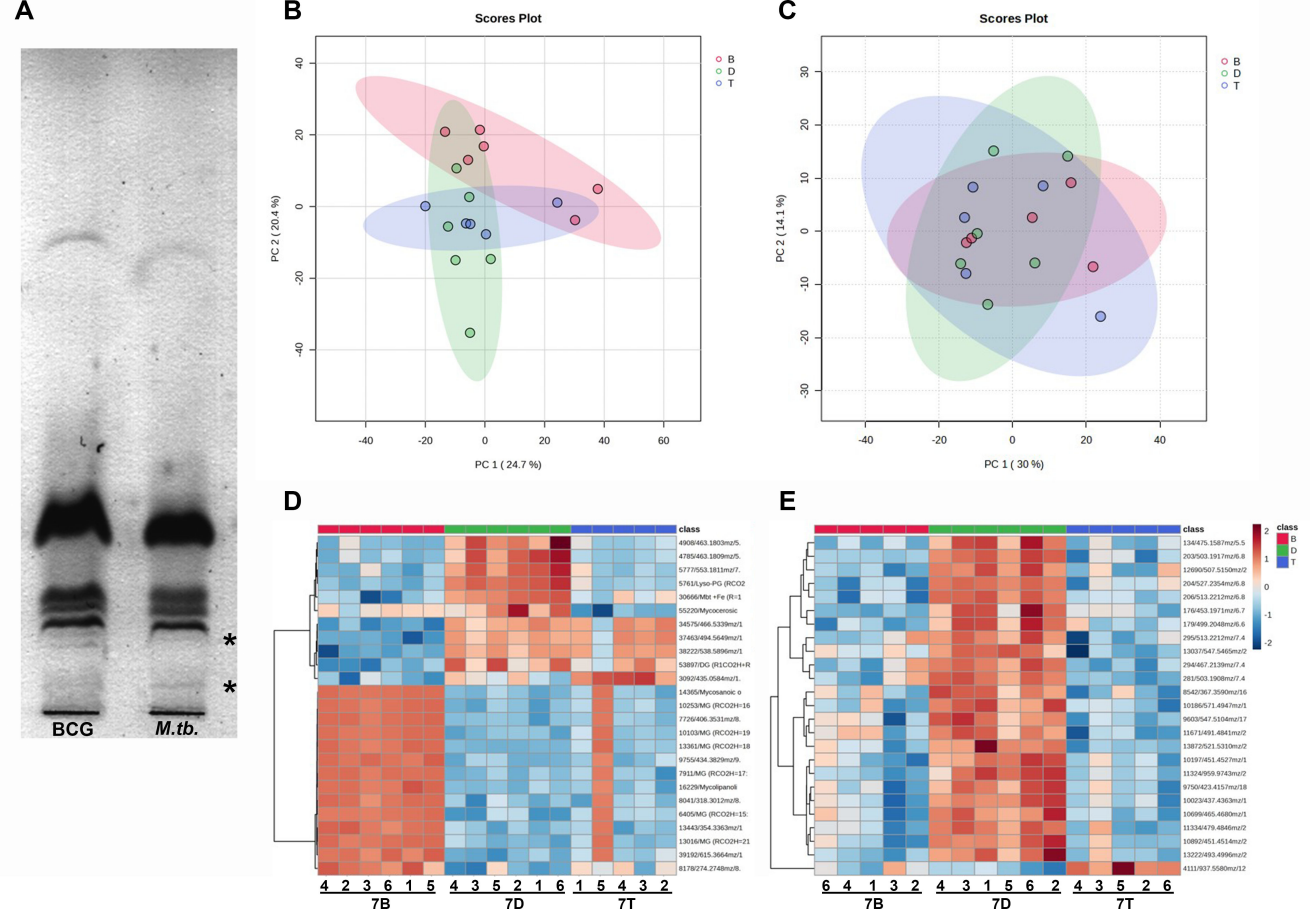
Impact of the *rv1371/2/3* operon on the lipid profile of *M. bovis* BCG Moreau. (**A**) TLC plate showing differential bands (*) near the origin of sample loading after a negatively charged molecule enrichment protocol corroborates that this operon may be involved in negatively charged lipid production. PCA analysis with feature data from the 5 replicates of Δ*rv1371/2/3*::BCG (B-red), *rv1371/2/3* knockout (D-green), and Δ*rv1371/2/3::M.tb*. (T-blue) strains in the (**B**) positive and (**C**) negative modes. Heatmaps after hierarchical clustering peak data from the (**D**) positive and (**E**) negative modes.

Negatively charged lipid-enriched samples from wild-type BCG Moreau complemented with its own allele and that of *M. tuberculosis* were initially analyzed by thin layer chromatography (TLC). As observed in Fig. 5A, there are differential bands near the sample loading origin, suggesting a more hydrophilic nature of these molecules.

Further LC-MS analysis was performed with crude extracts from *rv1371/2/3* knockout (Δ*rv1371/2/3*) and complemented (Δ*rv1371/2/3::M.tb*. and Δ*rv1371/2/3*::BCG) strains (*N* = 5). As can be observed by PCA analysis, results from the positive mode (Fig. 5B) were able to better discriminate strains when compared to the data from the negative mode (Fig. 5C). Furthermore, after hierarchical clustering peak data, the generated heatmaps with the first 25 features show that there are some features able to differentiate complemented strains from knockout in positive (Fig. 5D), but better in the negative mode (Fig. 5E). Aside from that, in positive mode results, there are some features distinguishing BCG Moreau-complemented strain from the one complemented with *M. tuberculosis*, allele, and knockout strains (Fig. 5D).

## DISCUSSION

Previous studies in the literature describe the products of *rv1372* and *rv1373* as an alpha-pyrone polyketide synthase, Pks18 (19), and a glycolipid sulfotransferase (18), respectively. Polyketide synthases (PKSs) are essential enzymes in microbial physiology and bear mechanistic and structural similarities to fatty acid synthases (FASs), although they often lack the reductase domain, resulting in partially reduced molecules. These enzymes are classified into three main types: type I PKSs are large multimeric complexes, where each polypeptide chain contains multiple enzymatic domains; type II PKSs are also multimeric, but each monomer has a single enzymatic function; and type III PKSs, often homodimeric, encapsulate all catalytic steps within a more compact complex (20). Type III PKSs, such as *M. tuberculosis* Pks18, are well-documented in plants, where they contribute to secondary metabolite production, particularly among members of the chalcone synthase superfamily. A notable feature of PKSs is their product diversity, producing compounds ranging from long-chain pyrones, as observed in Pks18, to mycocerosic acid and various secondary metabolites across fungi, bacteria, and plants. Unlike FAS, PKSs typically utilize pre-existing lipids as substrates to modify and extend carbon chains. Consequently, PKS activity relies on FAS products or the acquisition of external lipid chains. Rather than merely serving as auxiliary proteins, PKSs introduce structural diversity to standard fatty acid-derived molecules from FAS, thus expanding the functional range of these molecules (21).

The crystal structure of Pks18 (19) reveals that this enzyme possesses an unusual 20 Å substrate-binding tunnel, which may be associated with its broad substrate specificity for aliphatic long-chain acyl-coenzyme A starter units (C6–C20). Additionally, *M. tuberculosis* contains two other type III PKS genes, *pks11* and *pks10*, both organized in an operon structure, with PKS11 also reported to utilize long-chain starters (22). During catalysis, these enzymes extend the starter unit through sequential condensation with malonyl-CoA. Due to the absence of full reducing capability, the final products retain a pyrone moiety; in Pks18, this leads to a cyclic structure formed through intrachain reactions.

The glycolipid sulfotransferase activity attributed to the *rv1373* product is considered essential for bacterial development (11). Characterized through recombinant protein assays, *M. tuberculosis* Rv1373 functions as a glycolipid sulfotransferase, acting on typical ceramide glycolipids and mycobacterial trehalose glycolipids, potentially contributing to the sulfolipid IV biosynthetic pathway (18). The function of *rv1371* remains unconfirmed through activity assays; however, automated annotation based on homology suggests it may function as a fatty acid desaturase.

Thus, our initial hypothesis is that this entire operon encodes enzymes involved in lipid synthesis and modification. In this context, Pks18 would contribute to the production of the lipid core, while the glycolipid sulfotransferase and fatty acid desaturase would modify this core. However, we do not exclude the possibility that these gene products could also participate in other pathways, producing or modifying distinct lipids and/or other hydrophobic molecules. Accordingly, we hypothesize that due to the *rv1371* mutation in BCG, which results in a truncated C-terminus and possibly a translational uncoupling event between *rv1371* and *rv1372*, BCG Moreau may possess a nonfunctional sequence compared to the *M. tuberculosis* allele. This hypothesis is based on the overlap between the stop codon of *rv1371* and the start codon of *rv1372*, suggesting a translational coupling arrangement (23). Consequently, the frameshift caused by an insertion in *rv1371* could abolish or reduce *rv1372* expression. Supporting the impact on *rv1373*, there is another insertional mutation in this gene in BCG Moreau, which also results in a predicted truncated protein and may help explain the near-absence of sulfolipids in BCG strains (18).

Western blot analysis indicates that both genotypes express the two Rv1371 isoforms, corresponding to the shorter BCG product (33 kDa) and the *M. tuberculosis* “wild-type” protein (55 kDa). This may be attributed to population heterogeneity, allowing both native and mutated proteins to be detected in a multicellular assay, mimicking gene expression noise, as previously described (8). For Rv1373, only the *M. tuberculosis* isoform was detected in both bacterial strains. Given that the mutation occurs in a homopolymeric sequence (a string of seven consecutive guanines), this could be explained by a transcriptional or translational slippage event, where RNA polymerase or the ribosome, respectively, “stutters,” leading to both sequences being read similarly and thus producing identical final products. This correlation between coding sequence indels and increased slippage events is believed to confer an advantage by restoring “native” protein structure and activity, typically among other variants (24). Further assays are necessary to determine why only the “native” band was detected in the BCG Moreau sample, without a mixture of this isoform and the expected 29 kDa variant.

Another intriguing observation is that the detection of Rv1373 supports the possibility that this gene is not translationally coupled with *rv1371/2*, as suggested by sequence analysis showing a five-nucleotide gap between the stop codon of *rv1372* and the start codon of *rv1373*, reducing the likelihood of a coupling event. Thus, the distinct band patterns observed on TLC may reflect a partially or fully inactive operon in BCG Moreau due to the expression of an inactive Rv1371 and the possible suppression of Rv1372 (the lipid core-producing Pks18). In this context, Rv1373 may remain functional, as evidenced by detection of the *M. tuberculosis* isoform in BCG Moreau, and could act on other cellular substrates.

Regardless of the origin of these differences, it is important to emphasize that the lipid profile observed in BCGM::*rv1371/2/3M.tb*. correlates with significant changes in the growth curve of BCG Moreau Δ*rv1371/2/3::M.tb*. within J774 macrophages, as well as with the internalization rate during our infection assays. Interestingly, these alterations in cellular lipid content do not affect colony morphology, suggesting that the operon product does not act as a structural cell wall lipid. Nonetheless, it seems to interfere in the production of molecule(s) affecting cell permeability, as *M. tuberculosis* allele enhances survival after SDS exposure. This observation raises two nonmutually exclusive mechanistic possibilities. One possibility is that the operon-produced enzymes generate low-abundance lipidic or metabolic compounds that modulate cell envelope permeability or surface-exposed properties, thereby indirectly affecting bacterial uptake and intracellular fitness, without producing clear morphological changes. Alternatively, the proteins encoded by the *rv1371/2/3* operon may themselves exert regulatory or interaction-mediated functions, modulating lipid metabolism through protein–protein interactions rather than through the accumulation of a specific end product. Notably, the increased resistance to SDS exposure observed with the *M. tuberculosis* allele supports a model in which the operon impacts cell envelope permeability rather than bulk lipid composition. While our current data do not allow us to unequivocally distinguish between protein-driven and metabolite-driven mechanisms, they favor subtle biochemical or biophysical alterations of the cell envelope. Nonetheless, this distinct infection phenotype strongly indicates that the products of the *M. tuberculosis rv1371/2/3* operon—whether protein or, more likely, metabolites generated by these enzymes—impair the initial stages of infection while enhancing the bacterium’s performance in the host cell at later stages of interaction.

Several lipids and other virulence factors have been characterized in *M. tuberculosis* as facilitators of uptake by immune cells, with most, such as lipoarabinomannan (LAM), promoting bacterial internalization and thus contributing to a more successful infection (25). In this case, we identify an operon that produces a factor that negatively impacts this early infection stage, which may seem disadvantageous when solely considered. However, as evidenced by our other findings, this factor also enhances bacterial persistence within macrophages at later stages, potentially providing an advantage that outweighs the initial lesser negative impact. The loss of this factor in the *M. bovis* lineage may be associated with an early step in bacterial *in vitro* attenuation.

Furthermore, our shotgun lipidomics data using LC-MS indicate that it was possible to identify characteristic features for each analyzed strain (the knockout and the strains complemented with the *M. tuberculosis* or Moreau alleles). Analyzing the molecules in both positive and negative modes, we detected a group of features capable of distinguishing the complemented strains from the knockout (more pronounced in the negative mode), corroborating the idea that even with the polymorphisms identified in the Moreau allele, the operon appears to be partially active in this strain, generating a profile similar to that of the strain complemented with the *M. tuberculosis* allele. However, additionally, in the positive mode, there are features capable of differentiating the strain complemented with the Moreau allele from the other two strains. Once again, these facts support the notion that the products of this operon may participate in more than one metabolic pathway, causing each of the analyzed strains to behave as a point on a gradient of activity, with the knockout being inactive, the one complemented with the *M. tuberculosis* allele being the wild-type, and the Moreau strain being intermediate. Even though it was not possible to identify a singular or set of molecules responsible for the phenotypic differences observed in the SDS survival and macrophage infection assays, such preliminary findings indicate the importance of this operon, as well as demonstrate that the polymorphisms in Moreau do not completely inactivate this genetic system, further supporting the involvement of these products in more than one metabolic pathway.

With this set of data, we believe we can contribute to the discussion regarding the function of this operon in *M. tuberculosis* and other mycobacteria. Our Western blot analysis shows that despite the mutation, the *rv1373* product retains the *M. tuberculosis* “wild-type” structure, supporting the idea that the partial nonfunctionality of the BCG operon, as observed in TLC and infection assays, is primarily due to the alteration in Rv1371 and possibly the loss of Rv1372 expression, given its possible translational coupling with the former. Further assays focusing on the *rv1372* locus are necessary to confirm its role in the observed phenotype. Regardless of the exact source, we observed a difference in the infection process, suggesting that a factor produced by the *M. tuberculosis* operon, likely a negatively charged lipid, hinders the initial macrophage-bacterium interaction and internalization, yet aids mycobacterial growth during later stages of infection in our model. To further characterize this differential effect, we plan to use a mouse infection model to gain insights into the operon’s role in a systemic context, aiming to clarify its relevance to disease progression. This could also allow us to assess the potential of these genes as targets for tuberculosis drug development.

## MATERIALS AND METHODS

### Sequence analysis and comparisons

The *M. tuberculosis* H37Rv *rv1371/2/3* operon sequence (NC_000962.3, 154,3359..1,546,992) was retrieved from the NCBI public domain databank. The corresponding sequence for *M. bovis* BCG Moreau was identified through BLAST analysis, revealing BCG_M1398/1402 (1,541,472..1,545,107) on the *M. bovis* BCG strain Moreau RDJ complete genome (GenBank AM412059.2). Nucleotide and protein alignments were conducted using the Clustal Omega tool, available online.

### Qualitative RT-PCR for co-transcriptional analysis

Total RNA was extracted from *M. bovis* BCG Moreau cells using the TRIzol method followed by Turbo-DNase treatment. cDNA synthesis was performed using 1 μg of total DNA-free RNA with the SuperScript III First- Strand Synthesis kit (Invitrogen), employing random primers. RT-PCR amplification targeted the *rv1371–rv1372* and *rv1372–rv1373* intergenic regions, using the following primer pairs, respectively: TGTGTCGGAAGTATCTGTTG and AAGGCCAACCTCATGCCTCT, for 71–72 and TAGAGACGATGGTGCAGCAG and CGGCGCCGAGATAATGATGT for 72–73. Amplified products were analyzed by 1.0% agarose gel electrophoresis, with visualization achieved through ethidium bromide staining.

### Recombinant protein production, purification, and polyclonal sera production

For recombinant protein production, sequences corresponding to *M. bovis* BCG Moreau’s *rv1371* (1,541,472..1,542,422) and *rv1373* homologs (1,544,129..1,544,920) were cloned into the pET28a(+) vector, incorporating a C-terminal 6-His tag. Genomic DNA from *M. bovis* BCG Moreau was used as a template for PCR amplification, adding an NcoI restriction site at the 5′ end and an XhoI site at the 3′ end, using Invitrogen Platinum SuperFi DNA polymerase.

Following recombinant plasmid construction and sequence verification by colony PCR and Sanger sequencing in *Escherichia coli* TOP10, the plasmid was transformed into *E. coli* BL21(DE3) for protein expression, induced with 1 mM isopropyl-β-D-thiogalactopyranoside (IPTG) for 3 h at 37°C, 200 rpm in Luria-Bertani medium supplemented with the appropriate antibiotic.

His-tagged proteins were purified via Immobilized Metal Affinity Chromatography (IMAC) on a HisTrap HP 1 mL column (GE Healthcare) charged with Ni²^+^. Proteins were eluted using a step-gradient (10%, 20%, 30%, 40%, and 100%) of elution buffer (same composition as loading buffer [100 mM Tris-HCl pH 7.5, 300 mM NaCl, and 5 mM imidazole], but containing 500 mM imidazole). Purified proteins were used to immunize BALB/c mice, and the resulting sera were pooled to obtain polyclonal antibodies against Rv1371 and Rv1373 (α-Rv1371/Rv1373).

### Native protein in-gel characterization

*M. tuberculosis* H37Rv and *M. bovis* BCG Moreau cells were cultivated in 7H9 medium supplemented with 10% ADC (albumin, dextrose, and catalase) at 37°C under shaking (200 rpm). Cultures were initiated at an OD_600_ ~ 0.1, and cells were harvested by centrifugation on days 2, 5, and 7, corresponding to the lag, logarithmic, and stationary growth phases, respectively. After cell lysis and centrifugation, total protein concentration was determined by NanoDrop, and 20 μg of the protein was resolved on 15% SDS-PAGE, followed by Western blotting using polyclonal sera. Briefly, the nitrocellulose membrane containing proteins was incubated with specific primary antibody (1:1,000) for 2 h, followed by incubation with HRP-coated goat anti-mouse IgG (1:10,000) for 1 h. Following each antibody incubation, the membrane was washed three times with TBS-T, followed by three washes with TBS. All antibody incubations were performed in TBS containing 5% skim milk. Detection of the blots was carried out using the SuperSignal kit (Pierce) in accordance with the manufacturer’s instructions.

### Creation of *rv1371/2/3* knockout in *M. bovis* BCG Moreau and axenic growth analysis

A homologous recombination-based recombineering approach was used to replace the *rv1371/2/3* sequence from *M. bovis* BCG Moreau (1,541,472..1,545,132) with a kanamycin resistance gene (*kanR*). Approximately 1 kb regions upstream (1,540,471..1,541,471) and downstream (1,545,133..1,546,102) of the excised sequence, as well as *kanR,* were amplified by PCR, using the following primer pairs: TTGGATAACAAAGGCTGAACAT and ATTGTTCATGTGGTATTCCTCCAGGTCTTTC for 1 kb UP; AGGAATACCACATGAACAATAAAACTGTCTGC and GCAGCAACTGCATGAATTAATTCTTAGAAAAACTC for *kanR*; TTAATTCATGCAGTTGCTGCACTTTAGACG and TCCGCAGACCCTAATACAC for 1 kb DOWN. These sequences were further fused to construct the allelic exchange substrate (AES). The latter was electroporated into acetamide-induced pJV53H-harboring *M. bovis* BCG Moreau electrocompetent cells, with recombinants selected on 7H11 plates supplemented with 10% of a mixture of oleic acid, albumin, dextrose, and catalase (OADC) and kanamycin (40 μg/mL). Confirmation of the locus structure was performed by PCR using primers flanking the excised region and kanR, as well as sequences upstream and downstream of the target region.

Complementation was achieved by cloning the *rv1371/2/3* sequences from *M. bovis* BCG Moreau (1,541,475..1,545,119) and *M. tuberculosis* H37Rv (1,543,362..1,547,004) into the pMV361 vector (26), using the pBlaF promoter for heterologous transcription. For BCG Moreau complementation, *kanR* plasmids were used, whereas *hygR* was the one for *Drv1371/2/3* knockout complementation. Electrocompetent *M. bovis* BCG Moreau wild-type and knockout strains were transformed, and recombinants were selected on kanamycin/hygromycin (50 μg/mL) plates.

Axenic growth analysis was conducted on wild-type, *rv1371/2/3* knockout (Δ*rv1371/2/3*), and complemented strains (Δ*rv1371/2/3::M.tb*. and Δ*rv1371/2/3*::BCG), with cultures initiated at an OD_₆₀₀_ ~ 0.1 in 7H9 medium supplemented with 10% ADC. Growth was monitored by turbidometry analysis.

### Survival after SDS exposure

The SDS survival assay was conducted as previously described (27). Briefly, Δ*rv1371/2/3*, Δ*rv1371/2/3::M.tb*., and Δ*rv1371/2/3*::BCG strains were grown in 7H9 medium supplemented with 10% ADC and kanamycin (50 μg/mL). Mid-log phase cultures were diluted in fresh, antibiotic-free media to an OD_600_~ 0.05. For each strain, a control and a test culture were prepared, with the latter receiving a sterile 10% (wt/vol) SDS solution to achieve a final SDS concentration of 0.05% (wt/vol). Following incubation for 4 h at 37°C with mild agitation (100 rpm), serial dilutions were plated on 7H10 medium containing the necessary antibiotics for CFU counting. All experiments were performed in triplicate.

### Macrophage infection assays

J774 murine macrophage cells were maintained in RPMI 1640 medium supplemented with 10% fetal bovine serum and 1% amino acids 50× solution (Sigma Aldrich). For infection, 2 × 10⁵ macrophages were incubated with bacterial strains at a multiplicity of infection (MOI) of 10:1 for 4 h, followed by three washes with RPMI medium. The cells were then incubated at 37°C with 5% CO₂. Bacterial samples were collected at various time points (4, 6, 24, 48, 72, and 96 h) by differential lysis with 0.05% SDS. Following centrifugation, serial dilutions of bacterial suspensions were plated on 7H10 medium supplemented with 10% ADC for CFU counting after 3 weeks of incubation at 37°C.

### Statistical analysis

Data are presented as the mean ± standard deviation of three independent experiments. Statistical significance was determined using one-way or two-way ANOVA, with GraphPad Prism 5 software.

### Lipidomics analysis

Lipidomics analysis was performed using TLC and MS. For the first assay, *M. bovis* BCG Moreau strains complemented with their native or *M. tuberculosis* alleles were used. For MS analysis, strains Δ*rv1371/2/3*, Δ*rv1371/2/3::M.tb*., and Δ*rv1371/2/3*::BCG were used. Cells were cultivated under previously described conditions, except that Tween 80 was omitted from the medium, and cultures were maintained without agitation for 4–6 weeks. Total lipid extraction was conducted using a methanol/chloroform (2:1) mixture, followed by extraction with the same solvents in a 1:2 ratio. The combined extracts were dried and washed with water. For TLC, this crude extract was further enriched for negatively charged lipid species, using a Sep-Pak Accell Plus QMA anion exchange column (37–55 μm particle size) following the manufacturer’s protocol. Final lipid fractions were subjected to TLC, employing a chloroform/methanol/water (65:25:4) mobile phase on pre-coated TLC plates (DURASIL-25, silica gel 60, Macherey-Nagel). Lipid bands were visualized by CuSO₄/H₃PO₄/methanol staining, followed by heating at 100°C.

For MS analysis, the crude lipid extracts were used as samples (*N*≥5). Dried samples were solubilized in 200 µL chloroform/isopropanol 1:4 and then analyzed by liquid chromatography Nexera X2 UHPLC (Shimadzu), Zorbax Eclipse XDB-C18 column 4.6 × 50 mm 1.8u coupled to maxis Impact (Bruker Daltonics) mass spectrometry. The samples were run in positive and negative modes. For the positive mode, solvent A was 0.1% formic acid with 3 µL injection volume, and for the negative mode, solvent A was 5 mM ammonium acetate at pH 5 with 20 µL injection volume. For both conditions, isopropanol was used as solvent B. Injected samples were eluted at 50°C from the column at 0.3 mL/min with a binary gradient from 0% to 80% solvent B: 0.1–10 min, 80% B; 10–25 min, 80% B; 25–27 min, 10% B; 27–35 min, 10% B. Mass spectrometer parameters were as follows: nebulizer pressure 5 bar, dry gas 8 L/min, dry temperature 220°C, capillary voltage 4,500 V, end plate offset −500 V, quadrupole low mass 100 *m/z*, mass range 100 to 2,000 *m/z,* and 3 Hz in the DDA mode. The 5 most abundant precursor ions were selected for fragmentation with 30–60 collision energy.

Raw data files were converted to mzXML using DataAnalysis 6.1 (Bruker Daltonics). Data were analyzed using MZMine 4.0.3 (28). Lipid Annotation tool (28, 29) and Mtb Database (30) were used for lipid identification. Statistical analysis was done with MetaboAnalyst 6.0 (31).

## Data Availability

The mass spectrometry raw data (.mzXML files) generated in this study have been deposited to the MassIVE repository (https://massive.ucsd.edu) and are available under the data set identifier MSV000099769.
